# Prevalence, associated factors, awareness, treatment, and control of hypertension: Findings from a cross sectional study conducted as a part of a community based intervention trial in Surkhet, Mid-western region of Nepal

**DOI:** 10.1371/journal.pone.0185806

**Published:** 2017-10-05

**Authors:** Mahesh Kumar Khanal, Raja Ram Dhungana, Pratiksha Bhandari, Yadav Gurung, K. N. Paudel

**Affiliations:** 1 Ministry of Health, Bheri Zonal Ayurveda Hospital, Surkhet, Nepal; 2 Nepal Public Health Foundation, Kathmandu, Nepal; 3 Ministry of Health, Mid-western Regional Hospital, Surkhet, Nepal; 4 Population Services International Nepal, Lalitpur, Nepal; Medizinische Universitat Innsbruck, AUSTRIA

## Abstract

**Background:**

Hypertension is one of the leading public health problems globally. About half of the deaths from cardiovascular diseases were attributed to hypertension in 2008. Reduction of blood pressure to normal range is one of the major challenges in preventing complications and future burden of cardiovascular diseases. Therefore, this study aims to determine prevalence, awareness, treatment and control of hypertension and its associated factors in Nepal.

**Methods:**

This was a community based cross-sectional study conducted as a part of a community based intervention trial in Birendranagar Municipality of Surkhet district located at the Mid-western region of Nepal. We enrolled 1159 subjects aged 30 years and above. Out of 12 wards (administrative unit), four wards were selected randomly. Three hundred participants were recruited from each selected ward. Trained enumerator collected socio-demographic, anthropometric, and clinical data using standard STEPS questionnaires.

**Results:**

Out of all participants, women were 71% and mean age was 47±12.6 years. The overall prevalence of hypertension was 38.9% (95% CI: 36–41.7) while age and sex adjusted prevalence was 40.6%. The hypertension was present in 48.1% (95% CI: 45.2–50.9) of men and 35.2% (95% CI: 32.4–37.9)] of women. Male gender (OR = 1.49), older age (OR = 1.04 per year), Dalit caste (OR = 1.71), past history of cigarettes smoking (OR = 2.78), current alcohol consumption (OR = 1.75), and raised body mass index (OR = 1.17 per unit) were identified as significant factors associated with hypertension. Of total hypertensive respondents, 53.4% (95% CI: 48.7–58) were aware, 29% (95% CI: 24.8–33.1) were receiving treatment for high blood pressure, and 8.2% (95% CI: 5.6–10.7) had controlled blood pressure. The awareness, treatment, and control status were worse in younger participants.

**Conclusions:**

The study revealed high prevalence with low awareness, treatment, and control of hypertension in Nepal. Gender, age, ethnicity, smoking, drinking alcohol, and body mass index were associated with hypertension. Immediate public health and individual measures are warranted to reduce future burden of cardiovascular diseases.

**Trial registration:**

ClinicalTrial.gov (NCT02981251)

## Introduction

Hypertension is one of the major risk factors of cardiovascular diseases and one of the leading causes for global mortality [1]. Cardiovascular diseases accounted for one third of the total deaths a year in 2008. Out of these, 9.4 million deaths every year throughout the world were attributed to hypertension [2]. If left uncontrolled, hypertension is responsible for 45% deaths due to heart disease and 51% deaths due to stroke. In addition, the raised blood pressure can cause chronic kidney disease, heart failure, diabetes, and other diseases [3]. Globally, about 22% of adults aged 18 years and above had been diagnosed with hypertension in 2014. Prevalence was higher in low income countries compared to high income countries (30% in low income countries vs. 18% in Americas). The WHO estimated proportion of adults with high blood pressure was approximately 25% in South East Asia Region (SEAR) [4].

Nepal is one of the low income countries where nationally representative sample revealed that 25.7% of population aged 15–69 years suffered from hypertension in 2013 [5]. Similarly, the proportion of hypertension among different populations of Nepal were 12.3% in rural Sindhuli [6], 27.7% in Dhulikhel [7], 28% in Pokhara [8], 32.5% in Kathmandu [9] within last three years. Despite the high prevalence, control of hypertension was low in Nepal and ranged from 11.7% [7] to 24% [10].

Evidence shows that lowering and controlling blood pressure has health benefits. For instance, 10 mm Hg reduction of systolic blood pressure is associated with 22% and 41% reduction in coronary heart disease and stroke, respectively [11]. In the same way, prevention of different risk factors of hypertension contributed to 41–46% of the reduction in cardiometabolic mortality [12]. Population data on prevalence, awareness, risk factors, treatment, and control are necessary for establishing the gap, planning, and implementation of strategies related to hypertension prevention and control in communities. However, on one side, a very few studies have been conducted, on the other side, these studies were carried out only in the central and eastern part of Nepal [7,10] and scarce information is available in the Mid-western region. Therefore, this study was conducted to determine prevalence, associated factors, awareness, treatment and control of hypertension in Mid-western region of Nepal.

## Methods

### Study design, setting, and study population

This was a cross-sectional study conducted as a part of a community based health education intervention trial (trial registration no: NCT02981251) in Birendranagar Municipality of Surkhet District of Nepal from January to December 2016. The city is an administrative and geographic centre of the Mid-western part of Nepal, about 600 kilometers west of the capital where 52,137 population are residing in poor urban planning setup [13]. The city is inhabited by multicultural, multiethnic indigenous, and migrated population. This mixed population would be related to internal mobility (17% out of total migration of the region) of the Mid Western region [14]. These heterogeneous people are residing in each ward of the municipality [13]. Agriculture is the main source of income among the study population, followed by self-owned business and employment.

Men and women aged 30 years and above were included in the study. However, pregnant women and individuals who were bed-ridden, blind or deaf, diagnosed with kidney disease, cancer, heart disease, and mental illness were excluded from the study. As this study was the part of preventive health education program, we included the participants with essential hypertension and without complications.

### Sample size and sampling technique

Based on 36.8% prevalence of hypertension among the population of 30 years and above [15] with the precision of 5% and the design effect of 1.5, we calculated unadjusted sample size as 530. We used design effect to address the limitation (loss of power of the study) while applying clustered sampling compared to simple random sampling [16].The obtained sample size was doubled to incorporate two strata of men and women. After adjustment for a 10% non response rate, the final estimated sample size was 1179. We used multistage clustered sampling technique where wards (smallest administrative unit) were considered as clusters. Out of 12 wards, four wards were selected randomly. Initially, we randomly selected the first household from the left-hand side of the main road. Data collector, then, visited the consecutive households to enroll 300 participants, who met the inclusion criteria, from the single ward. We repeated the same sample selection strategy in each ward.

### Data collection

We recruited 20 health professionals and trained them about the study tools and methods of data collection. Principle and co-investigators organized three days training session for the data collector followed by pre-testing to conform their understanding. Training sessions covered study objectives, methods, inclusion and exclusion criteria, process of inform consent, accurate application of data collection tools, and techniques. Data enumerator interviewed face-to-face and observed anthropometric and blood pressure measurements of the study participants to collect the data. We utilized WHO STEPS questionnaires [17] to collect information related to age, sex, socio-economic status, tobacco use, alcohol consumption, fruit and vegetable intake, salt intake, and physical activities. The data collector asked participants to provide the information regarding total teaspoons of salt consumed during last two days. In addition, we asked whether they had ever been told by health worker that they have raised blood pressure or hypertension and whether they were receiving treatment for hypertension in the previous two weeks before the data collection. Similarly, other questions were related to information of the history of known diabetes mellitus and the family history of hypertension. Questionnaires were pre-tested among 20 participants before final implementation.

#### Blood pressure measurements

Trained data collectors measured the blood pressure using aneroid sphygmomanometer on the left arm. Appropriate cuff size was utilized based on upper arm circumference of the respondent. First reading was taken at least after 15 minutes of rest in uncrossed leg. Second measurement was obtained after the subject has rested for at least three minutes of the first measurement. If two readings were different, mean of two readings was used for final analysis [17,18].

#### Demographic and socio-economic variables

Age was recorded in completed years and re-grouped into ten years interval. Divorced, widow or widowers were regarded as single besides married and unmarried condition for marital status. Ethnicity was categorized into Brahman, Kshetri, Shahi or Thakuri, Dalit, and Janajati based on caste. Education was categorized into no formal education (illiterate/informal education), primary (grade 1–5), secondary (grade 6–10), and higher (grade >11) level. Occupation was classified as employed (involved in job or running own business), household work, agriculture or construction worker, and unemployed. Poor economic status was defined as any participant whose family income per person per year was less than Rupees 16, 355 (US$: 16.3) [19].

#### Hypertension awareness, treatment, and control variables

Hypertension was defined as average systolic blood pressure (SBP) ≥ 140 mm Hg and/or average diastolic blood pressure (DBP) ≥ 90 mm Hg and/or history of taking antihypertensive medication in the last two weeks [18]. Awareness of hypertension was defined as self reported condition of hypertension that had been diagnosed by a doctor or other health worker prior to the data collection [20]. Treatment of hypertension was defined as self reported use of any antihypertensive medication in last two weeks before the interview. Control of blood pressure was defined as SBP < 140 mm Hg and DBP < 90 mm Hg among hypertensive participants [20].

#### Variables related to behavior

Questions related to smoking use were asked to identify current, past use along with the number of cigarettes they were smoking per day. Current smoker was defined as those who were smoking cigarettes and those who quit less than one month before the interview. Similarly, those who were chewing tobacco in last 30 days were defined as current tobacco users [17]. Any respondent who drank alcohol within last 30 days of data collection was defined as current alcohol user. Total amount of alcohol intake was calculated in number of standard drinks (10 grams of pure ethanol). Number of servings of fruit and vegetable intake in a week was calculated with the help of pictogram. Sufficient intake of fruit and vegetable was considered if the participant consumed at least 400 grams or at least five servings per day [17]. Salt intake was estimated by dividing total teaspoons of salt by total family members who shared food in last two days. The average teaspoon was multiplied by 5.69 grams to get the total amount of salt intake. Dietary salt intake of >5 grams was considered as high amount of salt [21].Amount of physical activities was calculated in metabolic equivalents of task (MET) per minutes per week. They were categorized into light (<3 MET), moderate (3–6 MET) and vigorous (>6 MET) with the local context and questions were asked to record for each subject. Physical activities equivalent to less than 600 MET-minutes per week was defined as inadequate level (physically less active) [17].

#### Anthropometric measurements

Height and weight were measured by portable stadiometer and weighing machine. Waist circumference (WC) was measured in a separate room in standing position, at the end of a normal expiration, holding the arms relaxed at the sides, and at midpoint between the lower margin of the last palpable rib and the top of the iliac crest (hip bone).Weight was read in nearest kilogram (Kg) while height and waist circumference were measured in centimeter (cm). All measurements were undertaken in light clothes and without the shoes. Weight was divided by square of height (in meters) to calculate body mass index (BMI) of participants. The BMI was classified as underweight (<18.5 Kg/m^2^), normal (18.5–24.9 Kg/m^2^), overweight (≥25 Kg/m^2^), and obese (≥30 Kg/m^2^) [22]. Central obesity was defined as increased waist circumference more than 88 cm in women and more than 102 cm in men [23].

### Data management and analysis

Data were compiled and edited to maintain consistency before entering into Epidata V 3.1. Duplications were removed and exported to SPSS V.20.0 for further analysis. Simple descriptive statistics were used to describe socio-demographic characteristics of study participants. Data were presented as the frequency with percentage (%) for categorical variables and the mean with standard deviation (SD) for normally distributed continuous variables. Median with interquartile range was used to present non-normally distributed continuous data. The overall prevalence of hypertension was described for the total study population with 95% confidence intervals (CI). Initially, univariate analysis was used to identify factors associated with hypertension. Prevalence of hypertension and factors associated with hypertension were adjusted by age and sex using standard Asian population distribution of 2013 for both male and female [24]. Then all the associated factors were entered into simple and multiple logistic regression models to determine crude and adjusted odds ratios, respectively. Awareness, treatment, and control of hypertension were presented in proportion with 95% confidence interval for sex, age group, and socio-economic status among all hypertensive participants. Chi-square and independent t-test were conducted to compare proportions of categorical variables and means of continuous variables, respectively. Mann-Whitney U test used to test for medians of non-normally distributed numerical variables. All tests were two tailed and p value of < 0.05 was considered to be statistically significant.

### Ethical consideration

The study protocol was approved by the Ethical Review Committee of Nepal Health Research Council. Written informed consent was obtained from every participant along with thumb impression, if participants were unable to write. The study objectives, data collection procedures, benefit, risk, and confidentiality were explained to each respondent before obtaining consent.

## Results

### Socio-demographic characteristics of the study participants

Total number of participants included for the study was 1159 after excluding 21 with incomplete and inappropriate information. Of these, 71% were female. Mean age was 47±12.6 years and about one third of the participants were from 30–39 years. The majority of the enrolled residents were married. Out of total respondents, Brahman (29.3%) and Dalit (21.4%) were more compared to Janajati (18%). Majority of participants were not able to read and write (54%) and women (62.5%) were more uneducated than men (33.1%). More than half (56.8%) of the participants were working in household work while one third (26.3%) were employed. About one third (27.3%) were living in poor economic status (Table 1).

**Table 1:** Demographic, socio-economic, behavioral, and anthropometric characters of study subjects by sex.

Variable	Men (N = 335)	Women (N = 824)	Total (N = 1159)	
	n (%)	n (%)	n (%)	P value
Age (years)				
30–39	68 (20.3)	315 (38.2)	383 (33.0)	
40–49	91 (27.2)	204 (24.8)	295 (25.5)	
50–59	81 (24.2)	137 (16.6)	218 (18.8)	0.001
≥ 60	98 (28.4)	168(20.4)	263 (22.7)	
Marital status				
Unmarried	11 (3.3)	26 (3.2)	37(3.2)	
Married	314 (93.7)	711 (86.3)	1025 (88.4)	< 0.001
Single	10 (3.0)	87 (10.6)	97 (8.4)	
Ethnicity				
Brahman	100 (29.9)	240 (29.1)	340 (29.3)	
Kshetri	69 (20.6)	155 (18.8)	224 (19.3)	
Shahi or Thakuri	36 (10.7)	102 (12.4)	138 (11.9)	0.906
Dalit	71 (21.2)	177 (21.5)	248 (21.4)	
Janajati	59 (17.6)	150 (18.2)	209 (18.0)	
Level of education				
No formal education	111 (33.1)	515 (62.5)	626 (54.0)	
Primary (grade 1–5)	56 (16.7)	80 (9.7)	136 (11.7)	
Secondary (grade 6–10)	95 (28.4)	144 (17.5)	239 (20.6)	< 0.001
Higher (grade >11)	73 (21.8)	85 (10.3)	158 (13.6)	
Occupation				
Employed or self employed	163 (48.7)	142 (17.2)	305 (26.3)	
Household work	64 (19.1)	595 (72.2)	659 (56.8)	< 0.001
Agriculture or labor	81 (14.2)	61 (7.4)	142 (12.3)	
Unemployed	27 (8.1)	26 (3.2)	53 (4.6)	
Economic status				
Above poverty line	238 (71)	605 (73.4)	843 (72.7)	0.424
Poor	97 (29)	219 (26.6)	316 (27.3)	
Current Smoker	117 (34.9)	92 (11.2)	209 18(18)	
Past smoker	66 (19.7)	114 (13.8)	180 (15.5)	<0.001
Non smoker	152 (45.4)	618 (75)	770 (66.4)	
Current tobacco user	109 (32.5)	30 (3.6)	139 (12.0)	<0.001
Currently drinks alcohol	129 (38.5)	48 (5.8)	177 (15.3)	<0.001
Insufficient use of fruit and vegetable	320 (95.5)	802 (97.3)	1122 (96.8)	0.113
High salt intake (≥ 5 gm/day)	74 (22.1)	162 (19.7)	263 (20.4)	0.352
Inadequate level of physical activity	139 (41.5)	276 (33.5)	415 (35.8)	0.01
BMI				
Overweight	97 (29)	314 (39)	419 (36.1)	0.006
Obesity	15 (4.5)	72 (8.7)	87 (7.5)	
Central Obesity	44 (13.1)	415 (50.4)	459 (39.6)	<0.001
Mean±SD				
Age	50.87±12.39	45.5±12.4	47±12.6	<0.001
BMI	23.23±3.80	24.08±4.35	23.83±4.2	0.002
WC	90.80±12.78	87.52±14.47	88.47±14.08	<0.001
SBP	128.69±19.91	120.82±19.83	123.10±20.16	<0.001
DBP	83.86±12.85	79.64±12.02	80.86±12.41	<0.001

Note: Body Mass Index (BMI), Waist Circumference (WC), Systolic Blood Pressure (SBP), Diastolic Blood Pressure (DBP), Standard Deviation (SD)

### Prevalence of hypertension

**Fig 1:**
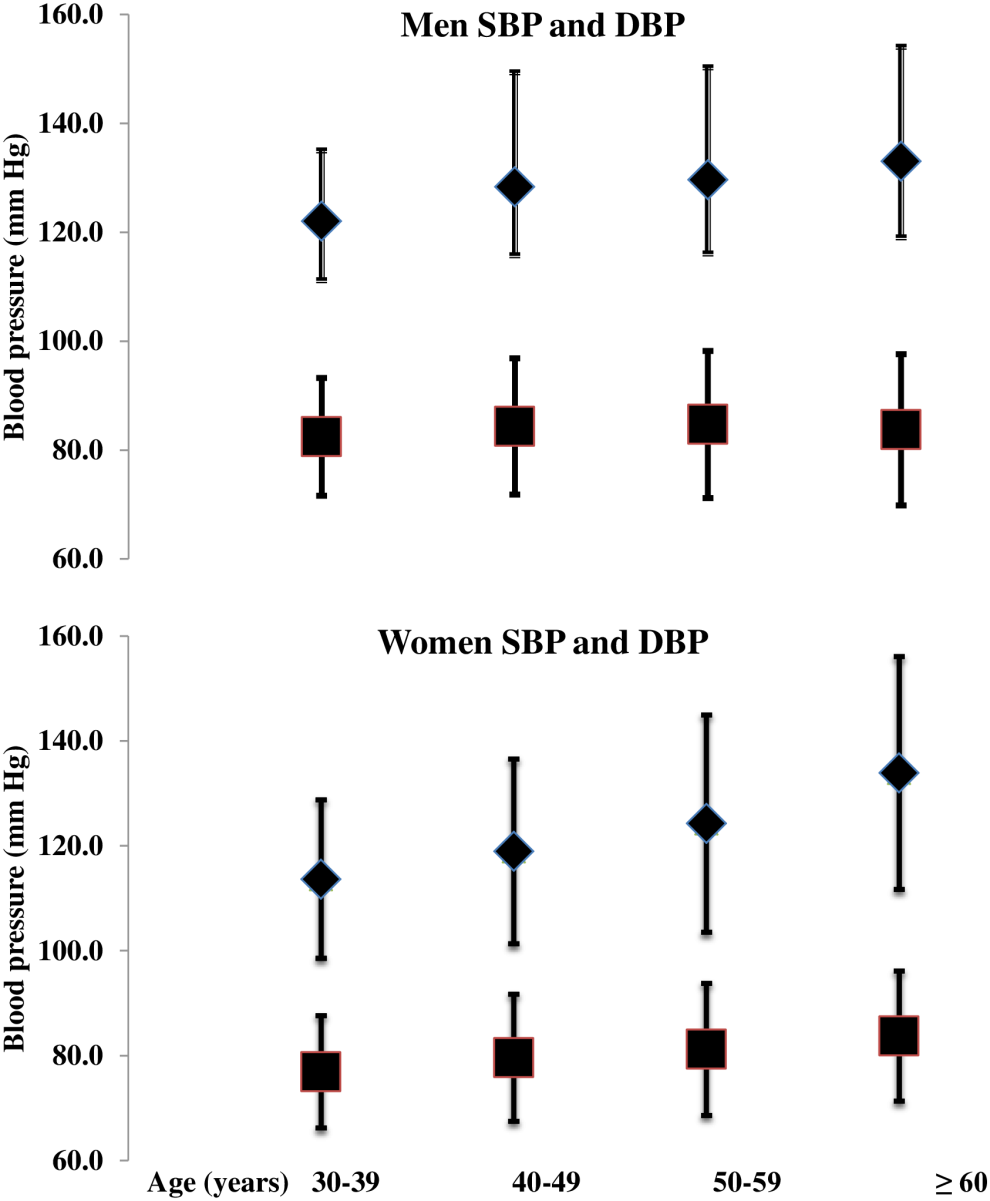
Mean SBP and DBP stratified by age and sex bars represent the standard deviation (SD), Abbreviations: SBP; systolic blood pressure and DBP; diastolic blood pressure.

**Fig 2:**
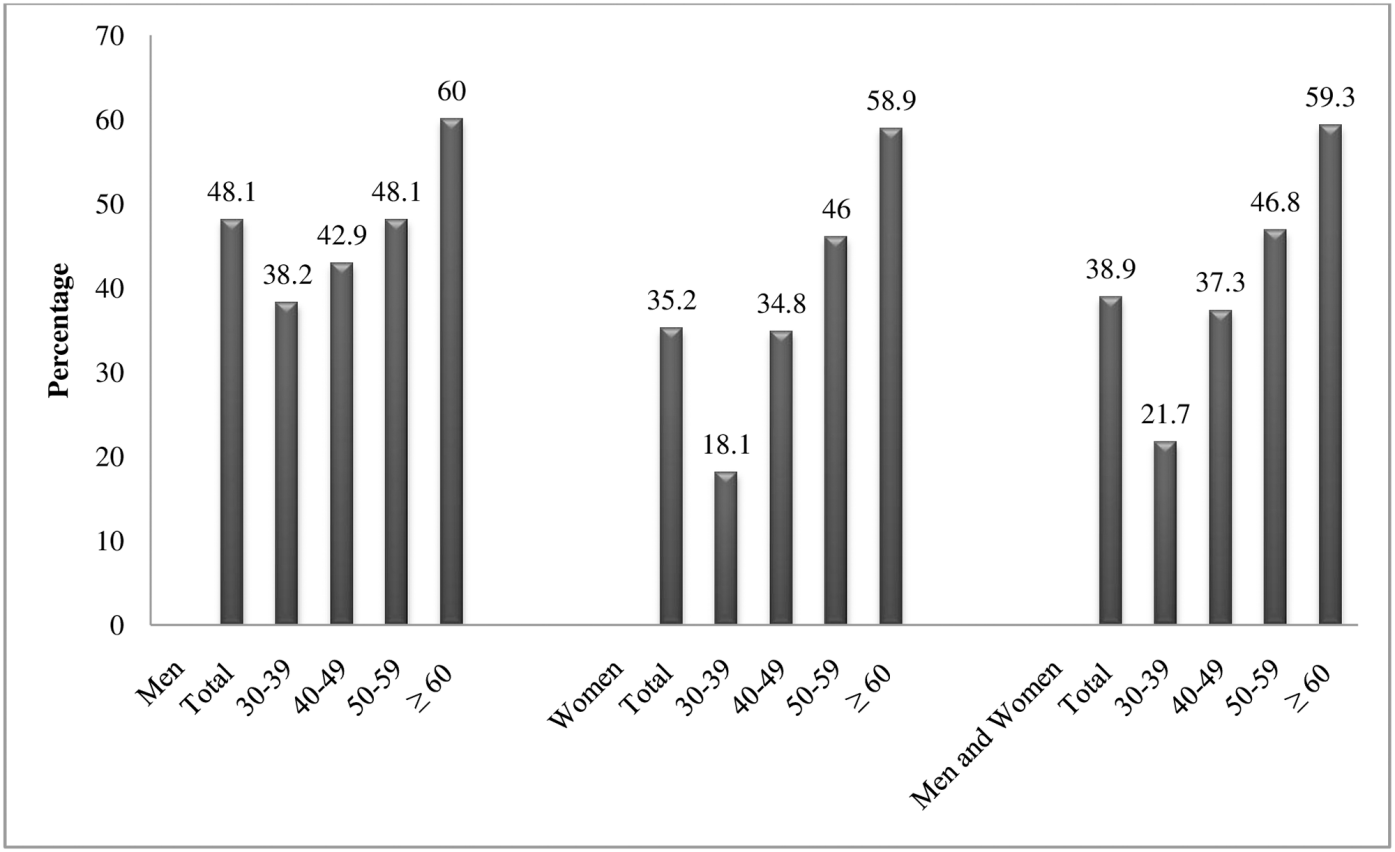
Prevalence of hypertension by sex and age.

Mean systolic blood pressure (SBP) was 123.1±20.6 mm Hg. The mean SBP was higher among men than women (128.6±19.9 vs. 120.8±19.8 mm Hg). Similarly, the overall mean diastolic blood pressure (DBP) was 80.8±12.4 mm Hg. The women (79.6±12 mm Hg) had lower mean blood pressure than that of men (83.6±12.8 mm Hg) [Table 1 and Fig 1]. After age and sex adjustment, overall mean systolic blood pressure was 123.6±20.1 mm Hg and mean diastolic blood pressure was 80.9±12.5 mm Hg. Men had significantly higher systolic and diastolic blood pressure compared to women (p<0.001).The overall prevalence of hypertension was 38.9% (95% CI: 36–41.7).With the age and sex adjustment hypertension prevalence was 40.6%. The prevalence of hypertension was significantly higher among men [48.1% (95% CI: 45.2–50.9)] compared to women [35.2% (95% CI: 32.4–37.9)]. After adjusting for age and sex, 48.6% of men and 37.1% of women were hypertensive. When stratified with the age group, the lowest prevalence was observed among 30–39 years of age (21.7%).The highest prevalence was 59.3% among participants above 60 years of age. The prevalence was almost similar among both men and women, if compared among last two age groups (Fig 2). Presence of hypertension was significantly different between men and women (p<0.001) and among age groups (p<0.001) with and without adjusting for age and sex (Table 2).

**Table 2:** Socio-demographic factors associated with hypertension.

Variable	Normotensive	Hypertensive	P value	Age sex adjusted P value
	n (%)	n (%)		
Sex				
Male	174 (51.9)	161 (48.1)	<0.001	<0.001
Female	534 (64.8)	290 (35.2)		
Age (years)				
30–39	300 (78.3)	83 (21.7)		
40–49	185 (62.7)	110 (37.3)		
50–59	116 (53.2)	102 (46.8)	<0.001	<0.001
≥ 60	107 (40.7)	156 (59.3)		
Marital status				
Unmarried	22 (59.5)	15 (40.5)		
Married	648 (63.2)	377 (36.8)	<0.001	<0.001
Single	38 (39.2)	59 (60.8)		
Ethnicity				
Brahman	222 (65.3)	118 (34.7)		
Kshetri	150 (67.0)	74 (33.0)		
Shahi or Thakuri	89 (64.5)	49 (35.5)	0.001	0.001
Dalit	124 (50.0)	124 (50.0)		
Janajati	123 (58.9)	86 (41.1)		
Level of education				
No formal education	336 (53.7)	290 (46.3)		
Primary (grade 1–5)	92 (67.6)	44 (32.4)		
Secondary (grade 6–10)	170 (71.1)	69 (28.9)	<0.001	<0.001
Higher (grade >11)	110 (69.6)	48 (30.4)		
Occupation				
Employed	185 (60.7)	120 (39.3)		
Household work	412 (62.5)	247 (37.5)	0.026	0.020
Agriculture or labor	89 (62.7)	53 (37.3)		
Unemployed	22 (41.5)	31 (58.5)		
Economic status				
Above poverty line	531 (63.0)	312 (37.0)	0.03	0.012
Poor	177 (56.0)	139 (44.0)		

### Socio-economic characteristics and hypertension

Hypertension was highest among the widow or widower (59%). Dalit (50%) and Janajati (41%) participants had high blood pressure in comparison to upper caste groups like Brahman and Kshetri. The proportion of hypertension was highest among uneducated participants (46.3%). Respondents without any job were more likely to be hypertensive. Prevalence was higher among those who had poor economic status (44%) compared to participants who were in above poverty line. Hypertension varied significantly with level of education (p = <0.001), marital status (p = <0.001), ethnicity (p = 0.001), occupation (p = 0.026), and economic status (p = 0.03) in univariate analysis. Moreover, ethnicity (p = 0.001), level of education (p = 0.001) and marital status (p<0.001), occupation (p = 0.02), and economic status (p = 0.012) were significantly associated with hypertension when adjusted for age and sex (Table 2).

### Behavioral and anthropometric factors associated with hypertension

#### Smoking

Overall, 18% and 15.5% of participants were the current and past smoker, respectively. Higher proportion of males (35%) than females (11%) were smoking cigarettes at the time of data collection. Among current smokers, 64.4% had high blood pressure. Mean number of cigarettes smoked was about 7 per week in both hypertensive and normotensive participants. Hypertension was observed among one third of past smokers. Smoking status (p<0.001) was significantly associated with hypertension with and without age and sex adjustment. Twelve percent of participants were chewing tobacco, among them; about half of the participants had hypertension. Current tobacco use (non smoking tobacco) was significantly (p = 0.01) associated with hypertension with and without hypertension (Tables 1 and 3).

**Table 3:** Behavioral and anthropometric factors associated with hypertension.

Variables	Normotensive	Hypertensive	P Value	Age sex adjusted P Value
	n (%) or Mean±SD or Median (interquartile range)	n (%) or Mean±SD or Median (interquartile range)		
Smoking				
Non smoker	115 (55.0)	94 (45.0)		
Current smoker	64 (35.6)	116 (64.4)	<0.001	<0.001
Past Smoker	529 (68.7)	241 (31.3)		
No of sticks per week	7.37±24.7	7.38±21.6	0.994	
Current tobacco use				
Yes	71 (51.1)	68 (48.9)	0.010	0.005
No	637 (62.5)	383 (37.5)		
No of packets per week	1.48±6.5	2.27±9.1	0.090	
Currently drinks alcohol				
Yes	77 (43.5)	100 (56.5)	<0.001	0.001
No	631 (64.3)	351 (35.7)		
Standard drink per day	3.00±1.55	3.65±2.25	0.032	0.020
Fruit and vegetable intake				
Sufficient	20 (54.1)	17 (45.9)	0.372	0.413
Insufficient	688 (61.3)	434 (38.7)		
Number of Servings	2.28 (1.17)	2.42 (1.71)	0.039*	
High salt intake (≥ 5 gm/day)				
Yes	148 (62.7)	88 (37.3)	0.566	0.427
No	560 (60.7)	363 (39.3)		
Level of physical activities				
Inadequate	224 (54.0)	191 (46.0)	<0.001	<0.001
Adequate	484 (65.1)	260 (34.9)		
Total METs-Minutes per Week	1590 (3375)	1060 (4040)	0.036*	
Diabetes mellitus				
Yes	30 (37.5)	50 (62.5)	<0.001	0.001
No	678 (62.8)	401 (37.2)		
Family history of hypertension				
Yes	91 (53.5)	79 (46.5)	0.020	0.037
No	617 (62.4)	372 (37.6)		
Body Mass index (BMI)				
Underweight	82 (76.6)	25 (23.4)		
Normal	417 (65.9)	216 (34.1)		
Overweight	176 (53.0)	156 (47.0)	<0.001	<0.001
Obesity	33 (37.9)	54 (62.1)		
BMI	23.07±3.99	25.03±4.27	<0.001	<0.001
Waist circumference (WC)				
Normal	466 (66.6)	234 (33.4)	<0.001	<0.001
Raised	242 (52.7)	217 (47.3)		
WC	86.12±13.14	92.16±14.7	<0.001	<0.001

*Mann-Whitney *U* test was used to compare non-normally distributed data.

#### Alcohol consumption

Proportion of participants who consumed alcohol currently was 15.3% where more than one third of males (38.5%) and 6% of females were drinking (Table 1). Majority of study subjects who drank alcohol (56.5%) had hypertension. Current alcohol consumption was associated with hypertension without (p<0.001) and with (p = 0.001) age and sex adjustment. The mean number of standard drinks per day was significantly (p = 0.032) higher among hypertensive respondents (3.6 vs. 3) (Table 3).

#### Fruit and vegetable intake

Almost all participants (96.8%) were not eating fruit and vegetable in sufficient amount and 38.7% of these individuals had hypertension. Median intake of fruit and vegetable servings was significantly different (p = 0.039) in both hypertensive and normotensive subjects (Tables 1 and 3).

#### Salt intake

Overall, one fifth of the total participants were consuming more than or equal to five grams of salt. Higher proportion of males (22.1%) was taking high amount of salt compared with females (19.7%). Of those participants who consumed high amount of salt, 37.3% had high blood pressure. However, salt intake was not significantly different between respondents with and without hypertension (Tables 1 and 3).

#### Physical activities

Out of all, 35.8% of participants were physically less active. Less physical activity was higher among males (41.5%) than females (35.8%) (Table 1). Among these less active individuals, 46% were suffering from hypertension. Median level of physical activities was 1060 METs-minutes per week among hypertensive while it was 1590 METs-minutes per week among normal participants (p = 0.036). The low level of physical activities was associated with hypertension with (p<0.001) and without (p<0.001) age and sex adjustment (Table 3).

#### Overweight and obesity

Proportions of total participants who were overweight and obese were 36% and 7.5%, respectively. Women were more overweight and obese (39% and 8.7%) compared to men (29% and 4.5%) (Table 1). Proportion of hypertension was significantly different (p<0.001) between respondents with and without overweight and obesity. The mean BMI (p<0.001) and waist circumference (p<0.001) were significantly different in between hypertensive and normotensive participants. The findings were significantly (p<0.001) different between hypertensive and normotensive participants even after adjustment with age and sex (Tables 1 and 3).

#### Diabetes mellitus and family history of hypertension

Seven percent participants stated that one of their first degree relatives had hypertension. Out of these, the proportion of hypertension was 46.5% among participants having positive family history and this family history of hypertension was significantly associated with hypertension with (p = 0.037) and without (p = 0.02) age sex adjustment. Overall, proportion of self reported diabetic participants were about 7%. It was significantly associated with hypertension without (p<0.001) and with (p = 0.001) age and sex adjustment (Table 3).

### Factors associated with hypertension

Univariate and multivariate logistic regression model were used to identify the factors associated with hypertension. Age, gender, marital status, ethnicity, level of education, occupation, economic status, smoking, alcohol consumption, level of physical activities, history of diabetes, family history of hypertension, and BMI were significantly associated with hypertension in univariate logistic regression. However, fruit and vegetable intake and consumption of high amount of salt were not significantly associated. When all of these variables were entered in a multivariate logistic model, only gender, age, ethnicity, smoking, current alcohol use, and BMI were identified as significant factors associated with hypertension. One year increase in age shifted the likelihood of having hypertension up by 4% (adjusted OR = 1.04, 95% CI: 1.03–1.06) after adjusting all other factors. The chance of having hypertension in males was 1.49 (95% CI: 1.00–2.22) times as compared with females. Odds of having hypertension in Dalits as compared to Bramhan was 1.71 (95% CI: 1.41–2.56). Similarly, among past smokers, the odds of having hypertension had increased by 2.78 (95% CI: 1.87–4.14) compared to non smoker. In the same way, current alcohol user had 75% (adjusted OR = 1.75, 95% CI: 1.13–2.71) more chance of having high blood pressure compared to non alcohol drinker. With every unit gain in BMI, the risk of having hypertension was increased by 17% (adjusted OR = 1.17, 95% CI: 1.13–1.21) even after all other factors were adjusted (Table 4).

**Table 4:** Multivariate analysis for factors associated with hypertension.

Variables	Crude Odds Ratio (95% CI)	Odds Ratio (95% CI)
Sex		
Female	Reference	Reference
Male	1.70 (1.31–2.20)**	1.49 (1.00–2.22)*
Age in years	1.05 (1.04–1.06)**	1.04 (1.03–1.06)**
Marital status		
Unmarried	Reference	Reference
Married	0.85 (0.43–1.66)	0.92 (0.43–1.99)
Single	2.27 (1.05–4.93)*	1.50 (0.61–3.66)
Ethnicity		
Brahman	Reference	Reference
Kshetri	0.92 (0.65–1.62)	0.86 (0.57–1.29)
Shahi or Thakuri	1.03 (0.68–1.56)	0.87 (0.53–1.41)
Dalit	1.88 (1.34–2.62)**	1.71 (1.41–2.56)*
Janajati	1.31 (0.92–1.87)	0.96 (0.63–1.46)
Education		
Higher (grade >11)	Reference	Reference
No formal education	1.97 (1.36–2.87)**	1.31 (0.79–2.17)
Primary (grade 1–5)	1.09 (0.66–1.79)	0.92 (0.52–1.65)
Secondary (grade 6–10)	0.93 (0.59–1.44)	0.89 (0.54–1.49)
Occupation		
Employed	Reference	Reference
Household work	0.92 (0.69–1.22)	0.97 (0.66–1.42)
Agriculture or labor	0.91 (0.60–1.38)	0.64 (0.39–1.06)
Unemployed	2.17 (1.20–3.93)*	1.47 (0.72–3.00)
Economic status		
Above poverty line	Reference	Reference
Poor	1.33 (1.02–1.73)*	1.14 (0.84–1.57)
Smoking		
Non smoker	Reference	Reference
Current smoker	1.79 (1.31–2.45)**	1.25 (0.84–1.86)
Past Smoker	3.97 (2.82–5.59)**	2.78 (1.87–4.14)**
Current alcohol consumption		
No	Reference	Reference
Yes	2.33 (1.68–3.23)**	1.75 (1.13–2.71)**
Physical activity		
Adequate	Reference	Reference
Inadequate	1.58 (1.24–2.02)**	1.26 (0.94–1.67)
Diabetes Mellitus		
No	Reference	Reference
Yes	2.81 (1.76–4.50)**	1.24 (0.73–2.11)
Family history of hypertension		
No	Reference	Reference
Yes	1.43 (1.03–1.99)*	1.17 (0.79–1.72)
Body Mass Index	1.12 (1.08–1.15)**	1.17 (1.13–1.21)**

* P value <0.05 and

** P value <0.001

### Awareness, treatment, and control of hypertension

**Fig 3:**
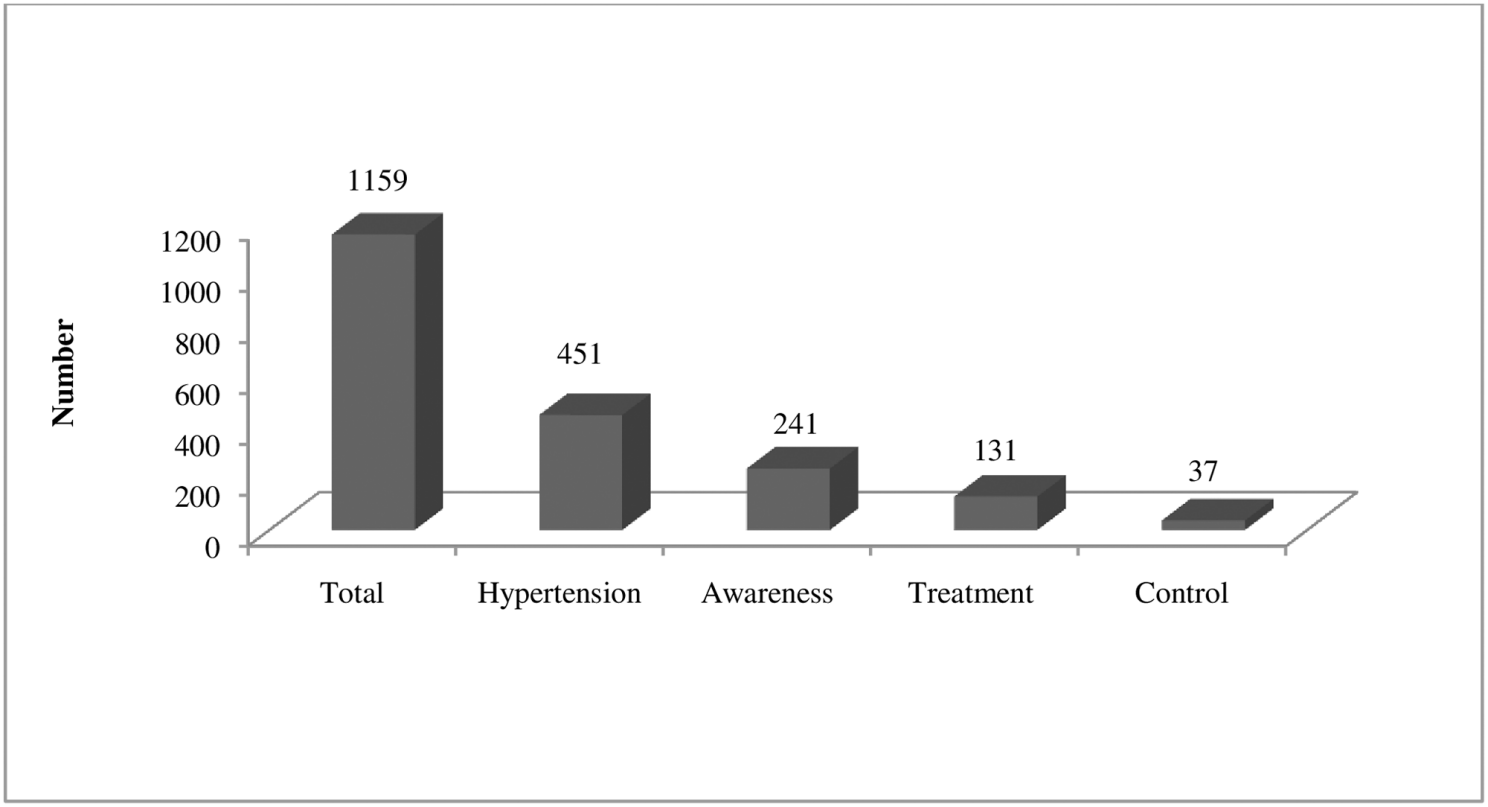
Summary of hypertension prevalence, awareness, treatment, and control.

Of total participants, 241 were aware of their hypertension, 131 were on treatment, and 37 had controlled blood pressure (Fig 3). The proportion of awareness among those who had hypertension was 53.4% (95% CI: 48.7–58). The awareness level was 55.9% (95% CI: 51.3–60.4) in males and 52.1% (95% CI: 47.4–56.7) in females. Older age groups had higher awareness of hypertension in both sexes compared to younger age groups. However, males more than 60 years were less aware than those of 40–60 years. Among those who had hypertension, 29% (95% CI: 24.8–33.1) were receiving prescribed antihypertensive medications. The proportion of men who were receiving treatment was 27.3% (95% CI: 23.1–31.4) and that of women was 30% (95% CI: 25.7–34.2) among hypertensive individuals. This treatment proportion was lower in younger age groups among all hypertensive participants. Only 8.2% (95% CI: 5.6–10.7) of all hypertensive participants had achieved blood pressure control (140/90 mm Hg). This achievement of blood pressure control was 7.5% (95% CI: 5–9.9) in males and 8.6% (95% CI: 6–11.1) in females. Blood pressure control was worse in younger individuals. Awareness (p = 0.003), treatment (p<0.001), and control (p = 0.039) were significantly different among the different age groups. However, the awareness, treatment and control among hypertensive participants were not significantly different by gender, ethnicity, level of education, occupation, and economic status (Table 5).

**Table 5:** Awareness, treatment, and control of hypertension among hypertensive participants at 95% confidence interval.

Variables	Awareness	Treatment	Control
	% (95% CI)	% (95% CI)	% (95% CI)
Overall			
Total	53.4 (48.7–58.0)	29.0 (24.8–33.1)	8.2 (5.6–10.7)
30–39 yrs	36.1 (31.6–40.5)	13.3 (9.9–16.2)	4.8 (2.8–6.7)
40–49 yrs	52.7 (48.0–57.3)	22.7 (18.8–26.5)	8.2 (5.6–10.7)
50–59 yrs	56.9 (52.3–61.4)	30.4 (26.1–34.6)	6.9 (4.5–9.2)
≥ 60 yrs	60.9 (56.3–65.4)	41.0 (36.4–45.5)	10.9 (8.0–13.7)
P values	**0.003**	**<0.001**	0.039
Men			
Total	55.9 (51.3–60.4)	27.3 (23.1–31.4)	7.5 (5.0–9.9)
30–39 yrs	26.9 (22.8–30.9)	0.0 (0.0–0.0)	0.0 (0.0–0.0)
40–49 yrs	64.1 (59.6–68.5)	28.2 (24.0–32.3)	10.3 (7.4–13.1)
50–59 yrs	64.1 (59.6–68.5)	25.6 (21.5–29.6)	2.6 (1.1–4.0)
≥ 60 yrs	57.9 (53.3–62.4)	40.4 (35.8–44.9)	12.3 (9.2–15.3)
P value	**0.011**	**0.002**	0.121
Women			
Total	52.1 (47.4–56.7)	30.0 (25.7–34.2)	8.6 (6.0–11.1)
30–39 yrs	40.4 (35.8–44.9)	19.3 (15.6–22.9)	7.0 (4.6–9.3)
40–49 yrs	46.5 (41.8–51.1)	19.7 (16.0–23.3)	7.0 (4.6–9.3)
50–59 yrs	52.4 (47.7–57)	33.3 (28.9–37.6)	9.5 (6.7–12.2)
≥ 60 yrs	62.6 (58.1–67.0)	41.4 (36.8–45.9)	10.1 (7.3–12.8)
P value	0.038	0.004	0.861
P values for men and women	0.434	0.549	0.665
Ethnicity			
Brahman	47.5 (38.4–56.5)	22.9 (15.3–30.4)	9.3 (4.0–14.5)
Kshetri	54.1 (42.7–65.4)	24.3 (14.5–34.0)	5.4 (0.25–10.5)
Shahi or Thakuri	63.3 (49.8–76.7)	36.7 (23.2–50.1)	16.3 (5.9–26.4)
Dalit	58.9 (50.2–67.5)	32.3 (24.0–40.53)	6.5 (2.1–10.8)
Janajati	47.7 (37.1–58.2)	32.6 (22.6–42.5)	7.0 (1.6–12.3)
P value	0.183	0.238	0.204
Education			
Illiterate/Informal education	55.5 (49.7–61.2)	32.1 (23.6–40.5)	8.3 (3.3–13.2)
Primary (grade 1–5)	59.1 (44.5–73.6)	29.5 (19.1–39.8)	4.5 (0.0–9.2)
Secondary (grade 6–10)	47.8 (36.0–59.5)	26.1 (13.8–38.3)	11.6 (2.6–20.5)
Higher (grade >11)	43.8 (29.7–57.8)	14.6 (8.3–20.8)	6.3 (2.0–10.5)
P value	0.289	0.091	0.556
Occupation			
Employed	52.5 (43.5–61.4)	23.3 (15.7–30.8)	4.2 (0.61–7.7)
Household work	52.6 (46.3–58.8)	31.2 (25.4–36.9)	10.1 (6.3–13.8)
Agriculture or labour	56.6 (43.2–69.9)	34.0 (21.2–46.7)	7.5 (0.4–14.5)
Unemployed	58.1 (40.7–75.4)	25.8 (10.3–41.2)	9.7 (0.0–20.1
P value	0.899	0.359	0.27
Economic status			
Above poverty line	51.9 (46.3–57.4)	28.2 (23.2–33.1)	8.7 (5.5–11.8)
Poor	56.8 (48.5–65.0)	30.9 (23.2–38.5)	7.2 (2.9–11.4)
P value	0.334	0.555	0.602

## Discussion

The current study revealed that about two-fifths of the participants had hypertension in suburban Municipality of Surkhet district of the Mid-western region of Nepal. Age, sex, ethnicity, smoking status, use of alcohol, and BMI were significantly associated with hypertension. Despite the high burden of hypertension, only half of them were aware of their condition, only one third were receiving the treatment, and one tenth had controlled blood pressure.

The prevalence of hypertension in the current study (38.9% and 40.6% without and with age/sex adjustment) was comparable with STEPS survey 2013 conducted in Nepal (36.8%) for respective age of 30 years and above [15]. The hypertensive patients were higher in the current study population as compared to other different population of Nepal such as the Eastern region (34%), the capital (32.5%),western region (28%), Dhulikhel (27.7%), and rural Sindhuli (12.3%) [6–9,25]. These disparities in findings may be due to an inclusion of younger participants (even up to 15 years) in other studies. The higher prevalence of hypertension observed among older age group in our study was consistent with other studies conducted in Nepal [25–27]. Our findings were also comparable to studies of India for instance the prevalence of raised blood pressure including those of antihypertensive drug user was almost same as that of Kerala (40%) state of India [28].

Age was significantly associated with hypertension and its prevalence was highest among participants aged more than or equal to 60 years (59.3%). One study conducted in Kathmandu Nepal revealed that 56.1% were hypertensive among the same age group [9]. Similarly, 45% of respondents aged more than 50 years had hypertension [26]. Arterial, arteriolar stiffness, increased sodium retention is related to rise of blood pressure with age [29].

Hypertension was significantly higher among men (48%) compared to women (35%) (OR:1.49) in our study. Age and sex adjusted prevalence of hypertension was 48.6% and 37.1% among men and women. The finding was consistent with latest STEPS survey which reported that odds ratio was 1.16 in males compared to females [5]. Similar findings were reported in the study conducted at Kathmandu (38.4% in males vs. 28.4% in females) [9], at that central development region (32.3% vs. 15.3%) [27] and at the rural Nepal (30.6% vs. 13.8%) [30]. Data synthesized from 46 population based studies of different countries found higher prevalence of hypertension in men than women [31]. One explanation for men to be more hypertensive may be related to the health behavior such as smoking, use of alcohol, and physical inactivity, which were consistently higher in male participants as noted from our study.

Ethnicity was another factor contributing to hypertension. Our results explored that Janajati had the highest burden of hypertension, which was followed by Dalit. Most of the studies conducted in Nepal found that hypertension was higher among the Janajati population. The higher exposure to unhealthy behaviors like smoking, use of alcohol among Dalits and Janajati population as observed in current and previous studies might be the contributing factors to hypertension [7,9,27]. However, more studies are needed to explain the effect of race/ethnicity on hypertension prevalence in Nepal.

In our study, current smoking and past smoking were associated with hypertension in univariate analysis. However, only past smoking became the significant predictor of hypertension when other variables were adjusted. Despite the well established fact that cigarette smoking increases the risk of hypertension [32,33], some cross-sectional epidemiological studies [34] like ours observed that habitual smokers generally have lower blood pressures than non smokers. This may be due to the fact that many patients stop smoking once they were diagnosed with hypertension.

Current alcohol consumption was also associated with hypertension. Recent epidemiological studies have demonstrated that alcohol intake was positively and significantly associated with risk of hypertension [35,36]. According to the global burden of disease in 2003, about 16% of all hypertensive diseases were attributed to alcohol consumption [37]. Different studies in Nepal also observed an association of alcohol consumption to hypertension [6,9]. However, some studies found protective effect of alcohol intake on blood pressure [38,39] and this effect was demonstrated in women with light and moderate alcohol consumption [35,36]. Our study found that mean standard drink was higher among hypertensive participants, which was consistent with a meta-analysis of 15 randomized controlled trials [40] that demonstrated a dose-response relationship between alcohol consumption and blood pressure [41].

Our study observed a positive association between body mass index and blood pressure, which was consistent with the findings from other studies conducted in similar settings in Nepal [6, 9]. Higher BMI means greater fat stores, insulin resistance, increased salt retention, [42,43] and decreased physical activity [44]; all these contribute to rise in blood pressure level.

Current study found that about half (53.4%) of all hypertensive participants were aware of their condition. The level of awareness in our study was higher when compared with other studies of Nepal and abroad. The proportion of aware participants ranged from 41% to 46% in Nepal [7,8,45]. Moreover, one cross-sectional study of 628 communities of high, middle and low income countries observed that 46.5% were aware of hypertension [20]. Our study also found that hypertension awareness was comparatively higher among elderly groups. It could be because of frequent visit to health care facilities by elderly groups. Similarly, one third (29%) of total hypertensive participants were receiving antihypertensive medicine in current study. This proportion was consistent with other studies of Nepal (31–33%) [7,8] and abroad (32.5%) [20]. However, the proportion of untreated hypertensive participants was less compared with a study in municipalities of Kathmandu [10] and then national STEPS survey of Nepal (88.3%) [15]. Current study contrast the finding of other studies which reported different awareness and treatment proportion among males and females [7,10]. As suggested by Devkota et. al., such a low treatment status could be because of poor socio-economic status, poor health literacy and ingrained misconception on antihypertensive treatment among participants [10]. In the same way, only 8.2% of all hypertensive participants had controlled blood pressure in current study which was lower than that reported in recent studies of Kathmandu (13%) [10], Dhulikhel (11%) [7], and Pokhara (15%) [8] of Nepal. When compared with the findings of developed countries, there was high disparity in awareness, treatment, and control of hypertension in Nepal [20,46]. Our results also showed hypertension control status significantly varied with age groups as observed in other studies [7,10]. From the above findings, it can be inferred that there is a huge gap in hypertension awareness, treatment and control in the study population inviting urgent public health intervention particularly targeting the population at risk, including younger population.

Our study had several limitations. The study was conducted in one municipality of the Mid-western region of Nepal with a total population of about 52, 000. High numbers of female were participated in the study because of the fact that many males were out of the community or country for earning livelihood. Though we had adjusted the sample size for non response rate, we did not record the formal participation rate. We have measured blood pressure twice at three-minutes interval in a single visit and average of these two were used for analysis. Over-estimation of hypertension status may result in due to the use of a single visit measurement. Moreover, bilateral measurement could rule out sublcavian steal syndrome. As we did not include the person having known heart disease and kidney disease in the study, findings could be less accurate in estimating population parameter. Despite the use of standardized questionnaire with rigorous training to the enumerator, error might have happened in self reported behavior. Over-estimation of level of physical activities and under-estimation of salt intake might have been observed in our study. It would have been better to use a log book or other direct method to ascertain physical activities [47] and 24-hours urinary sodium excretion to assess salt intake [48]. Presence of diabetes was determined as reported by participants without blood sugar measurement. This study could not measure cholesterol level also. Despite these limitations, this study has several strengths. This was the first study conducted in the Mid-western region of Nepal that reported the prevalence of hypertension by gender, age and socioeconomic status. Random selection of participants, rigorous training to data collectors, enrolment of the participants at their own homes, age-sex stratified analysis of data, and estimation of age-sex adjusted (by standard Asian population) prevalence of hypertension were other strengths of our study.

## Conclusions

This study found high prevalence but poor awareness, treatment, and control status of hypertension in the Mid-western region of Nepal. Age, sex, ethnicity, cigarette smoking, alcohol consumption, and body mass index were identified as associated factors of hypertension. Current findings suggest the future burden of cardiovascular and kidney diseases in Nepal. Immediate planning and implementation of public health measures and individual interventions are needed to prevent the occurrence and complications of hypertension.
